# The Importance of the Global Health Strategy from the U.S. Department of Health and Human Services

**DOI:** 10.4269/ajtmh.2012.12-0400

**Published:** 2012-09-05

**Authors:** Nils Daulaire

**Affiliations:** Office of Global Affairs, U.S. Department of Health and Human Services, Washington, District of Columbia

The world in which we live is more interconnected today than ever before. In an era when we can get anywhere on the planet in less than a day, and when the flow of persons and goods stretches worldwide, we must ensure that the systems for preventing, detecting, and containing disease stretch equally far. Likewise, we need to remain just as vigilant when it comes to safeguarding the food and drug products that go into our bodies.

For an illustration, let us look at Dulles Airport, a port of entry to the Washington, DC metropolitan region. In 2010, more than 6.4 million international passengers came through Dulles, and that is just one of three major airports in the area. Each day upward of one million persons drive across our borders, arrive at U.S. airports, or dock in our ports. What is more, we import half of the fruit and more than three-fourths of the seafood we consume, often from countries with far fewer safety controls than we enjoy here at home.

The notion that diseases or contamination somehow recognize geographic or political borders is a dangerous illusion. As my boss, Health and Human Services Secretary Kathleen Sebelius, often remarks, “We can no longer separate global health from America's health.”

Fortunately, the United States has a broad, diverse, and world-class range of experience and expertise in dealing with all manner of global health issues. Much of this work is performed within federal agencies throughout the government, through non-governmental organizations, and within research institutions. Although most work at my agency, the United States Department of Health and Human Services (HHS), is geared toward protecting and promoting the health of Americans domestically, we are no strangers to the global health environment. Each of our 11 agencies and 18 staff offices engages in global health activities, and we have placed more than 300 of our department's officials in more than 75 countries, providing on-the-ground guidance and input. Nor is global health a new area for HHS; we have been actively engaged in global health efforts for decades. As far back as 1948, HHS joined with the health ministries of other countries to create the World Health Organization (WHO), the health arm of the United Nations. Our scientists and epidemiologists have been at the forefront in the effort to eradicate smallpox; we invested heavily in tuberculosis, malaria, and human immunodeficiency virus/acquired immunodeficiency syndrome research, treatment, and prevention; and we have supported the development of new vaccines and treatments for neglected diseases of the world's poorest people. And our global health portfolio continues to grow.

With such a wide array of professionals and departments within HHS working on global efforts to prevent disease, promote health, and strengthen partnerships, we needed to find a way to pull together our work and bring it into a coherent whole. That is why Secretary Sebelius called for the creation of a unified department strategy for dealing with global health issues. In response, the Office of Global Affairs recently unveiled the HHS Global Health Strategy (GHS) at the beginning of 2012.

The GHS is the product of many months spent exploring the range of global health work being done within all of HHS; examining the rationale behind that work; identifying top priorities; and synchronizing the role of HHS in a comprehensive, cohesive strategy to guide us in developing programs that will have real impact and be mutually reinforcing. The strategy is based on several underlying principles, including using evidence-based knowledge to inform decisions; responding to local needs and building local capacity; and emphasizing prevention.

The strategy identifies 10 objectives into which we now have categorized the efforts within HHS on global health matters: the first three focus on surveying, preventing, and responding to outbreaks and disaster; the next two emphasize improving the safety of international supply chains and strengthening standards for food and drug and feed production; and two more encourage increased research and sharing best practices worldwide. The final three objectives look forward to the emerging challenges of the 21st century by encouraging global action to address the major current and emerging contributors to global death, illness, and disability; supporting President Obama's Global Health Initiative (GHI); and advancing health diplomacy.

By increasing our effectiveness in these 10 key areas, we aim to achieve, through global health action, three overall goals: protecting and promoting the health and well-being of Americans; providing leadership and technical expertise in science, policy, programs, and practice; and advancing United States interests in international diplomacy, development, and security.

So what exactly does HHS do in each of these objectives? In each instance, we are harnessing the power of what we already do very well throughout the agency while using the strategy as a roadmap to make sure we are maximizing dollars, impact, and results.

When thinking about the first cluster of three objectives, prevention, detection, and response, it is important to note that HHS is recognized globally for its unsurpassed technical expertise and experience in disease surveillance, notably through the Centers for Disease Control and Prevention. In concert with critical partners such as the U.S. Agency for International Development and the Department of Defense, we will continue to support countries and organizations to strengthen surveillance systems and make sure we are plugging gaps. We assist with improving workforce and laboratory capacity to support diagnosis for disease surveillance. And we ensure those surveillance efforts are timely, evidence-based, data-driven, and internationally shared and actionable to inform public health policies and decision-making.

When it comes to prevention, HHS agencies provide direct prevention-focused and preparedness-oriented support and technical support throughout the world. We support the development of sustainable capacities for addressing both public health emergencies and day-to-day public health needs. We facilitate the development, use, and evaluation of vaccines and other prevention strategies, such as vector control and safe drinking water. We support database and information technology infrastructure with global access for monitoring purposes, including early warning systems and monitoring holdings of dangerous pathogens. And we coordinate with global partners to disseminate public information and emergency notification when necessary.

In the event of public health emergencies, HHS is prepared and able to respond internationally as well as at home. We maintain a wealth of domestic and international scientific, technical, and operational expertise that is frequently called upon during and after international emergencies. We coordinate with others, including the U.S. Agency for International Development and WHO to investigate outbreaks, to treat the sick and injured, and to mitigate impact on people's lives. We support the development of sustainable response capacities and provide technical expertise in investigating disease outbreaks and identifying their cause. We collaborate with international partners to develop best practices for responding to natural and man-made disasters. And as laid out by the GHS, we will continue to develop policy frameworks, agreements, and operational plans to facilitate HHS decision-making in response to requests for emergency assistance.

With the second cluster of objectives, global supply chains and standards, the road forward is clear. Principally through the Food and Drug Administration, HHS works to enhance regulatory systems and global manufacturing and supply chains to ensure the safety of medical products, food, and feed. Our efforts help to identify key risks in the supply chain and we implement strategies to mitigate those risks in cooperation with other governments and international agencies. And we work to strengthen strategic regulatory partnerships that promote a safer, higher quality global supply of medical products, food, and feed.

Through our active membership in WHO and a range of specialized international agencies, HHS also continues to provide leadership to establish, strengthen, and implement science-based international health and safety standards. The agency supports multilateral efforts to improve global health policies, programs, and practices. To those ends, we work to ensure an appropriate leadership role for the United States in the development of science-based norms and standards. And we work to strengthen existing multilateral relationships while developing new strategic alliances to maximize the achievement of our global health goals.

Innovating biomedical and public health research and exchanging best practices globally are key to improving health and well-being across national borders. HHS places a strong emphasis on both of those areas and strives to accelerate scientific discovery to improve patient care. The National Institutes of Health serves as the global gold standard in biomedical research. Priorities identified within the GHS include addressing research linked to scientific opportunity, public health needs, and the evolving burden of disease. We support the rapid translation of research results into new or improved preventive, diagnostic, and treatment products and processes. And we encourage research that identifies causative pathways of the spread of infectious disease and other health threats.

HHS brings to the global health arena technical expertise in the areas of human resources for health, service delivery, and regulatory science and systems. We support collaborative health system strengthening activities, including workforce development. We promote global exchange of best practices and lessons learned to ensure that evidence supports decisions and program implementation. And we strive to address the underproduction and retention of health professionals in developing countries.

The final three objectives in the GHS position us for the future. As we move deeper into the 21st century, the global health challenges we face are changing. Shifting global disease burdens include cardiovascular disease, cancer, chronic respiratory illnesses, diabetes, environmental risk factors, and an aging population. In this area, HHS promotes the development, implementation, evaluation, and dissemination of cost-effective prevention, policies, and strategies for non-communicable diseases, just as we have done for decades for communicable diseases. We advance the integration of effective public health policies and trade policies. And we work to strengthen health system capacities to address the changing global patterns of death, illness, and disability by fostering evidence-based interdisciplinary practice and promoting integrated community approaches.

HHS and many of its agencies have participated actively in the development and implementation of the President's Global Health Initiative. The initiative is designed to improve health outcomes related to control of HIV/AIDS, TB, malaria, and neglected tropical diseases in low-income countries. GHI recently entered a second phase of the initiative, shifting the leadership of in country coordination from Washington D.C. to Ambassadors in the field. However the principles and design of the GHI remain the same, and countries continue to focus on the health of women, newborns, and children through programs for nutrition, safe water, and reproductive, maternal, and child health. HHS contributes to the achievement of GHI goals, focusing our efforts on county ownership and integration and coordination, health system engineering and the integration of public health services for prevention and control.

Finally, HHS currently works within the broader context of U.S. foreign policy to engage on health issues with diplomatic partners and to strengthen peer-to-peer technical, public health, and scientific relationships. The GHS outlines top priorities, including assigning health attaches to key U.S. embassies for international cooperation. We are working to establish a global health career track within HHS to formalize career opportunities and training for our staff working in global health, both domestically and internationally. We already partner with the Department of State to bolster knowledge about global health among the diplomatic corps. And we work to strengthen diplomatic knowledge, negotiation skills, and understanding of development principles for HHS field staff and technical health experts.

Although the GHS provides crucial coordination and guidance for HHS global health work, it also makes clear there is much to be done. Under each of the 10 objectives outlined above, there are a plethora of steps to be taken and critical work to do. My team, the Office of Global Affairs, is working on incorporating the GHS and its underlying principles into workplans throughout HHS, and we are prioritizing activities and partnerships while working with the entire U.S. government to improve coordination and communication.

To be certain, the Global Health Strategy is not an attempt to create an entirely new direction for HHS; rather it is meant to provide a clearer focus for our efforts in the coming years. We have ample evidence that no country can protect the health of its citizens alone. Our global health work is therefore not an addition to our efforts to improve health here in America, but rather a necessary extension of those efforts. Through robust cooperation with other nations and international organizations, we will continue to work to reduce the risks of disease, disability, and premature death for all our citizens, and in turn, all persons worldwide.

